# Proteomics Analysis of the Zebrafish Skeletal Extracellular Matrix

**DOI:** 10.1371/journal.pone.0090568

**Published:** 2014-03-07

**Authors:** Maurijn Y. Kessels, Leonie F. A. Huitema, Sjef Boeren, Sander Kranenbarg, Stefan Schulte-Merker, Johan L. van Leeuwen, Sacco C. de Vries

**Affiliations:** 1 Laboratory of Biochemistry, Wageningen University, Wageningen, the Netherlands; 2 Experimental Zoology Group, Wageningen University, Wageningen, the Netherlands; 3 Hubrecht Institute-KNAW and University Medical Centre Utrecht, Utrecht, the Netherlands; University of Sheffield, United Kingdom

## Abstract

The extracellular matrix of the immature and mature skeleton is key to the development and function of the skeletal system. Notwithstanding its importance, it has been technically challenging to obtain a comprehensive picture of the changes in skeletal composition throughout the development of bone and cartilage. In this study, we analyzed the extracellular protein composition of the zebrafish skeleton using a mass spectrometry-based approach, resulting in the identification of 262 extracellular proteins, including most of the bone and cartilage specific proteins previously reported in mammalian species. By comparing these extracellular proteins at larval, juvenile, and adult developmental stages, 123 proteins were found that differed significantly in abundance during development. Proteins with a reported function in bone formation increased in abundance during zebrafish development, while analysis of the cartilage matrix revealed major compositional changes during development. The protein list includes ligands and inhibitors of various signaling pathways implicated in skeletogenesis such as the Int/Wingless as well as the insulin-like growth factor signaling pathways. This first proteomic analysis of zebrafish skeletal development reveals that the zebrafish skeleton is comparable with the skeleton of other vertebrate species including mammals. In addition, our study reveals 6 novel proteins that have never been related to vertebrate skeletogenesis and shows a surprisingly large number of differences in the cartilage and bone proteome between the head, axis and caudal fin regions. Our study provides the first systematic assessment of bone and cartilage protein composition in an entire vertebrate at different stages of development.

## Introduction

The vertebrate skeleton is a specialized tissue that provides support and protection for other tissues, enables mechanical functions, and acts as a homeostatic mineral reservoir. The skeleton consists of bone and cartilage that is produced by two distinct cell types called osteoblasts and chondrocytes, respectively. The formation of skeletal elements is realized by two distinct modes called intramembranous (dermal) and chondral ossification. During intramembranous ossification, mesenchymal cells proliferate and differentiate into osteoblasts that produce bone matrix. During chondral ossification, the mesenchymal cells differentiate into chondrocytes that form a cartilage template. This initial cartilage template provides a stable scaffold for bone formation and enables growth of skeletal elements prior to complete ossification [1]. Chondrocytes first enter a maturation process, differentiating from small round cells into discoid proliferating chondrocytes that align into columns and regulate the growth of the cartilage element. Chondrocytes then enter a pre-hypertrophic phase during which they expand in volume and form fully differentiated hypertrophic chondrocytes. At this stage the chondrocytes secrete extracellular matrix. These hypertrophic chondrocytes then go into apoptosis, allowing for osteoblast precursors to migrate into the degrading cartilage matrix where they differentiate and deposit the bone matrix [2].

The extracellular matrices (ECMs) of bone and cartilage are mainly composed of a few highly abundant components. The major components of cartilage are the structural proteins of the heterotrophic collagen type II/XI/IX that comprises around 60% of the dry weight of cartilage [3]. The second largest group of structural proteins in cartilage (10–15%) is the proteoglycans. The most abundant proteoglycan is aggrecan that is responsible for the compression resistance of cartilage together with the heterotrophic collagens, and several other proteoglycans. In contrast, bone predominantly consists of a mineral fraction (50–70%) [4]. Additional to this mineral phase, the major component of bone is the structural protein collagen type I that comprises approximately 90% of the protein fraction in bone. During bone formation, collagen type I fibrils act as a scaffold for the growing bone minerals [5]. So-called non-collagenous proteins occupy the remaining 10% of the extracellular bone matrix. These non-collagenous proteins consist mainly of extremely acidic proteins which are believed to play crucial roles in the formation and function of mineralized tissues [6].

The mechanical properties of the skeleton are largely dependent on the composition of proteins that are secreted into the ECM. This protein diversity cannot be sufficiently derived from mRNA analysis (e.g. microarray techniques) since mRNAs are not inherently part of the ECM, and not all proteins that contribute to the formation of skeletal elements are produced in the vicinity of these elements. In addition, mRNA abundance has been shown to correlate poorly to the protein content [7], [8], [9], and does not take into account the wide variety of post-translational modifications which are critical to protein functions [10]. This makes proteomics an essential tool for characterizing the composition of the skeletal ECM. Progress in the field of proteomics in both technology and methodology allow for creating large datasets from complex samples with high mass accuracy and sequencing speed [11]. Mass-spectrometry (MS)-based proteomics is becoming more quantitative, now including protein quantification via label-free and stable isotope labeling technologies [12]. Proteomics analysis of bone and cartilage in human, mouse, and rat already provided valuable insights by investigating differential protein expression in bone and joints, articular cartilage, bone cells, and chondrocytes [13].

The zebrafish is an excellent system for proteomics since it is a well-characterized vertebrate model with readily available genetic maps allowing for the identification and characterization of proteins using existing databases. While being a relatively new model organism in the field of skeletal development, zebrafish have been used as a powerful model organism for the identification of novel gene functions during skeletogenesis [14]. With key regulatory genes being highly conserved between teleosts and other vertebrate species [15], zebrafish can provide important complementary information to studies performed in other species [16], [17]. At the molecular level, the biological similarity between zebrafish and humans is striking and has currently resulted in the identification and analysis of zebrafish genes homologous to human genes associated with disease [18]. In order to further our understanding of normal skeletal development, it is necessary to identify and characterize changes in skeletal composition, which in turn sheds light on the mechanisms that contribute to the production, functioning, and maintenance of the vertebrate skeleton.

Here, we present the first overview of the zebrafish skeletal proteome during development from larva to adult, using an MS-based approach. With a focus on proteins relevant for skeletal formation, 40 extracellular proteins were identified that are novel in the context of zebrafish skeletogenesis and with an implicated role in the development of the vertebrate skeleton.

## Materials and Methods

### Animal maintenance

Zebrafish wild type strains were reared at the fish facility of Wageningen University at 27.1°C with a 14:10 light dark cycle. All fish were raised in a density of 5 fish/L. For the experiments, matings were set up with two males and three females. Eggs were kept at 28.5°C at the breeding facility, and transferred to the fish facility at 20 days post fertilization (dpf). All fish were fed *ad libitum* three times a day. Zebrafish larvae were fed in-house cultured paramecium from 120 hours post fertilization. Between 10 and 60 dpf the fish were fed both paramecium and artemia, after which the paramecium was exchanged for Tetramin flakes. Zebrafish were kept under standard conditions until they reached 14, 28, or 358 dpf, at which point the fish were euthanized with 0.1% (w/v) tricaine methane sulphonate (TMS, Crescent Research chemicals, USA) buffered with 0.08% (w/v) sodium bicarbonate (Gibco, Paisley, Scotland).

### Ethic statement

The experiment was approved by the Wageningen University Animal Experiments Committee (protocol nr. 2011026.a and 2012020.a).

### Tissue preparation and extraction

Craniofacial, axial, and caudal fin skeletal elements were isolated from 500 zebrafish of 14 dpf, 100 zebrafish of 28 dpf, and 15 zebrafish of 358 dpf. First, the head region was separated from the axial skeleton between the head and the Weberian vertebrae. The axial skeleton included the Weberian vertebrae, the precaudal, and the caudal vertebrae [19]. The caudal fin was excised at the start of the caudal fin vertebrae. An overview of skeletal elements exhibited by the 3 developmental stages, obtained by acid-free bone and cartilage staining as described previously [20], is shown in Fig. S1. Skeletal structures were isolated from 14 day old larvae by removal of excess tissue using Accumax solution (Millipore). Larvae were incubated for 1 hour at room temperature while pipetting the sample up and down every 10 minutes using a syringe with a 23G needle. After 1 hour, the skeletal structures were collected using a 70 µm cell strainer (BD Falcon). The bone structures from 28 and 358 dpf zebrafish were excised manually, trimmed free of excess tissue and incubated in Accumax solution for 2 hours at room temperature under vigorous shaking to remove cell remnants.

### Protein extraction

For protein extraction the sequential protein extraction method developed by Jiang *et al*. [21] was adapted for optimal protein extraction from the limited sample sizes used here. The main modifications included the removal of the pre-extraction step in 4M guanidine-HCl (GdmCl) to prevent protein loss from the bone structures of zebrafish larvae that were minimal in their degree of ossification as compared to juvenile and adult bone structures. Secondly, the extraction volumes were kept to a minimum so that processing of the extracts could be performed using spin filters [22], [23].

Before protein extraction, the isolated material was washed in phosphate buffered saline (PBS) containing a protease inhibitor cocktail (Roche), pH 7.4, for several hours at 4°C. For the protein extraction, the material from the initially separated craniofacial and axial region was used to create triplicates containing 5 mg (wet weight) each. Skeletal material was transferred to 1.5 ml Eppendorf tube containing 6 stainless steel balls (diameter, 3.9 mm), snap frozen in liquid nitrogen, and pulverized using a Retsch mill (Retsch GmbH, Hann, Germany) for 3 times 15 seconds at 30 Hz. Since not enough skeletal material could be isolated from the caudal fin region of zebrafish larva (14 dpf), this sample point was excluded. The isolation from the juvenile (28 dpf) caudal fin region resulted in enough material for one sample only (5 mg), and was therefore extracted as one sample and measured in duplicate. After pulverization the samples were sequentially extracted in order to improve the protein extraction from the mineralized matrix of bone. (I) First, the samples were demineralized by incubation in 1.2 M HCl for 2 hours (larvae), or overnight (juvenile and adult) at 4°C. The supernatant was collected after centrifugation; the pellet was washed with PBS containing protease inhibitor cocktail (pH 7.4) and also collected after centrifugation. The HCl and PBS fractions were pooled and stored at –80°C. (II) The pelleted skeletal material was incubated in 50 mM Tris-HCl, 4M GdmCl, 0.5 M tetrasodium EDTA (pH 7.4) containing protease inhibitor cocktail, for 72 h at 4°C. The supernatant was collected after centrifugation, and the EDTA was removed by dialysis overnight in Slide-A-Lyzer mini dialysis units (Thermo scientific) against 50 mM Tris-HCl, 4 M GdmCl at 4°C. The dialyzed solution was subsequently stored at –80°C. (III) The remaining pellet was incubated for 24 h at 4°C in 50 mM Tris-HCl, 6 M GdmCl (pH 7.4) containing protease inhibitor cocktail. The solution collected after centrifugation was stored at –80°C.

### Filter-aided sample preparation

Filter aided-sample preparation was employed to prevent protein loss during the subsequent precipitation steps. Since two of the extraction steps were performed in high chaotropic salt (GdmCl) concentrations and all extraction steps were performed without a detergent like sodium dodecyl sulfate (SDS), the filter-aided sample preparation method allowed for the use of sample volumes up to 400 µl. To include all the proteins extracted in the first step (HCl extraction), the subsequent extracts of each sample were pooled to obtain a sample containing a high chaotropic salt concentration. Cysteine residues were reduced with 7.5 mM dithiothreitol (DTT) for 30 min at room temperature. 400 µl of the samples was added to a Pall 3K omega filter and centrifuged at 15,871 *g* for 30 min. The flow-through was discarded and this step was repeated until the complete sample volume was filtered. 400 µl 8 M urea in 100 mM Tris/HCl, pH 8 was added to the filter and centrifuged at 15,871 *g* for 30 min. Subsequently, 400 µl 0.05 M iodoacetamide/8 M urea in 100 mM Tris/HCl, pH 8 was added to the filter, mixed, incubated for 30 min in the dark at RT, and centrifuged at 15,871 *g* for 30 min. The filter was washed by the addition of 110, 120, and 130 µl 8 M urea in 100 mM Tris/HCl, pH 8 in subsequent steps with a centrifuge step in between each step, 15,871 *g* for 30 min. 140 µl 0.05 M NH_4_HCO_3_ (ABC) solution was added to the filter unit and centrifuged at 15,871 *g* for 30 min. This was then followed by the addition of 100 µl ABC solution containing 0.5 µg trypsin to the filter and incubated overnight at room temperature. A final centrifugation step was then performed at 15,871 *g* for 30 min and the flow through was acidified using 10% (v/v) trifluoroacetatic acid to pH 2-4. The samples were stored at –20°C until the nLC-MS/MS measurement was performed.

### Mass spectrometry measurement

The analysis was performed by injecting 18 µl of sample over a 0.10×32 mm Prontosil 300-5-C18H (Bischoff, Germany) pre-concentration column (prepared in-house) at a maximum pressure of 270 bar (Thermo Proxeon Easy nLC). The peptides were eluted from the pre-concentration column onto a 0.10×200 mm Prontosil 300-3-C18H analytical column with an acetonitrile gradient at a flow rate of 0.5 µl/min. The gradient that was used here increased the acetonitrile in water percentage from 9 to 21% during 100 min, 21 to 34% in 26 min, 34 to 50% in 3 min and constant at 50% for 5 min. Acetic acid was added to the eluent at 5 ml/l. The column was cleaned by increasing the acetonitrile up to 80% in 3 min. Between the pre-concentration and analytical column, an electrospray potential of 3.5 kV was applied directly to the eluent via a metal needle electrode fitted into a P777 Upchurch micro cross. Spectra were obtained between m/z 380 and 1400 on an LTQ-Orbitrap XL (Thermo electron, San José, CA, USA). MS/MS scans of the 10 most abundant doubly and triply charged peaks in the FTMS scan were recorded in data-dependent mode in the linear trap (MS/MS threshold =  5000, 60 s exclusion duration).

### Data analysis and label-free quantification

MaxQuant software version 1.2.2.5 was used to analyze the raw files from the LTQ-Orbitrap. MS/MS spectra were searched against the Uniprot zebrafish database downloaded from www.uniprot.org (2012), and a contaminant database including frequently observed contaminants such as human keratins and trypsin. For label-free quantification, the MaxQuant settings were kept default (using a 1% false discovery rate (FDR) on both peptide and protein level) except for the following changes. Asparagine or glutamine de-amidation was specified as variable modification. Label-free protein quantification was switched on, together with the “match between runs” option used with a maximal retention time window set to 2 min, and the intensity based absolute quantification (iBAQ) on. Normalized intensity values (LFQ intensity) were used for relative quantification. The MaxQuant results are shown in Table S1 in File S1. Filtering of the MaxQuant protein groups result file (Table S1 in File S1) was performed using the Perseus module (version 1.3.0.4). Reverse hits were removed from the data together with proteins with only 1 identified peptide, no unique peptide, or no unmodified peptide. Database search and quantification results were grouped by combining results for the different injections of the craniofacial, axial, and caudal fin region for each of the developmental stages.

Gene ontology (GO)-terms were specified for each protein using Software Tool for Researching Annotations of Proteins (STRAP) program analysis [24]. For genes with missing zebrafish ontology information, human orthologs were used instead and the same STRAP analysis was performed. Proteins known to be located in the (proteinaceous) extracellular region or matrix were used for further analysis. For quantification purposes, the proteins were additionally filtered and required to be detected in 2 or more out of each triplicate LC-MS/MS runs in at least 2 out of 3 sample points per region. Proteins without enough label-free quantification values were not selected for this procedure. Selected proteins were further analyzed using the Perseus module. Relative protein abundances were compared by performing a pair-wise t-test (both sides), with a permutation-based on FDR, threshold values of 0.05 or 0.01 and S0  =  1, with 250 number of randomizations and –log10 as selected parameters. The obtained protein ratios were used to assess differences in protein abundance between the different regions and developmental stages. Proteins that changed significantly in abundance were subsequently analyzed based on previous identification and/or characterization in the vertebrate skeleton by assessing literature and zebrafish-specific data available at ZFIN [25]. Proteins that passed the threshold value of 0.05 were explored by Ingenuity Pathway Analysis (IPA; Ingenuity Systems, Redwood City, CA). Protein abundance ratios (Table S5 in File S1) and corresponding t-test values were uploaded into IPA and used to predict biological processes differing in abundance during the complete time course from larvae to adult.

### In situ mRNA hybridization

Four of the proteins that differed in abundance (Table S5 in File S1) were selected for spatial gene expression analysis with in situ hybridization. Primers were designed using Primer 3 software [26] and extended with a T7, or Sp6 transcription initiation sequence on the reverse primer (Table S6 in File S1). cDNA obtained from zebrafish of 15 dpf was used to perform a PCR with these primers to generate the specific coding sequences of the genes (0.2–1.0 kb) to use as templates for the DIG labeling reaction. DIG-labeled probes were synthesized according to the manufacturer’s instructions (Roche, DIG-labeling) and purified with the Qiagen RNeasy kit (Protocol RNA Cleanup).

Wild type zebrafish (28 dpf) were euthanized as described above and fixed overnight in 4% paraformaldehyde (PFA) in phosphate-buffered saline (PBS) at 4°C. After washing with PBS (2×5 min), fish were dehydrated in 100% methanol at RT (room temperature, 2×5 min) and stored at –20°C until further use. For sectioning, fish were rehydrated (75%, 50% and 25% methanol/PBS, 1×5 min), and washed in PBST (PBS + 0.1% v/v Tween 20, 3×5 min). Fish were embedded in 1.5% agar/5% sucrose and incubated overnight in 30% sucrose at 4°C. Embedded fish were frozen in liquid nitrogen and stored at –80°C. Longitudinal sections of 20 µm were made with the cryostat (Leica CM3050S) and transferred onto polylysine coated slides (Menzel-Gläser, Thermo scientific). Slides were stored in a box with silica gel at –20°C or if used immediately, dried overnight at RT.

In situ hybridization was performed as described in Smith *et al*. [27] and Schulte-Merker [28] with the following modifications. Probes were diluted to a final concentration of 0.5 ng/µl in hybridization buffer (50% formamide, 5x salt solution; 0.75 M NaCl and 0.075 M sodium citrate pH = 7.0, 0.1% v/v Tween 20, 9.2 µM citric acid, 0.5 mg/ml tRNA yeast (Invitrogen), heparin (45 U/ml). Two hundred µl of the probe solution was added to each slide and covered with Nescofilm (Bando Chemical IND., Kobe, Japan). Slides were incubated overnight at 70°C in a humid chamber. Slides were washed in solution A (50% v/v formamide, 1 x salt solution, 0.1% v/v Tween 20), 2×30 min, 70°C) and in 1xTBST (0.14 M NaCl, 2.7 mM KCl, 0.025 M Tris HCl, 0.1% v/v Tween 20, pH = 7.5), 2×30 min, RT. Subsequently, slides were incubated in 2% blocking buffer (Blocking reagent, Roche, Mannheim, Germany) in 1 x TBST for at least 1 hour at RT. The blocking buffer was replaced with 200 µl of a 1∶2000 dilution of anti-DIG AP FAB fragments (Roche) in 2% blocking buffer. The slides were covered with Nescofilm and incubated overnight at 4°C. Then, slides were washed with TBST and equilibrated with AP staining buffer (0.1 M Tris, 0.1% v/v Tween 20, 0.1 M NaCl, 0.05 M MgCl_2_), 2×10 min. Slides were incubated in 400 µl of staining solution (1 ml AP staining buffer, 4.5 µl NBT and 3.5 µl BCIP) in the dark. Staining was monitored regularly and stopped by washing in distilled water (2×10 min). After clearing with methanol, washing with PBS and fixation with 4% PFA/PBS (20 min at RT), slides were mounted in Aquatex (Merck, Darmstadt, Germany) and incubated overnight at RT to dry. Images were taken with an Olympus DP50 digital camera mounted on a Nikon Microphot-FXA microscope and Analysis^D^ software (Soft Imaging System GmbH, Germany).

## Results

### Zebrafish skeletal extracellular proteome

**Figure 1:**
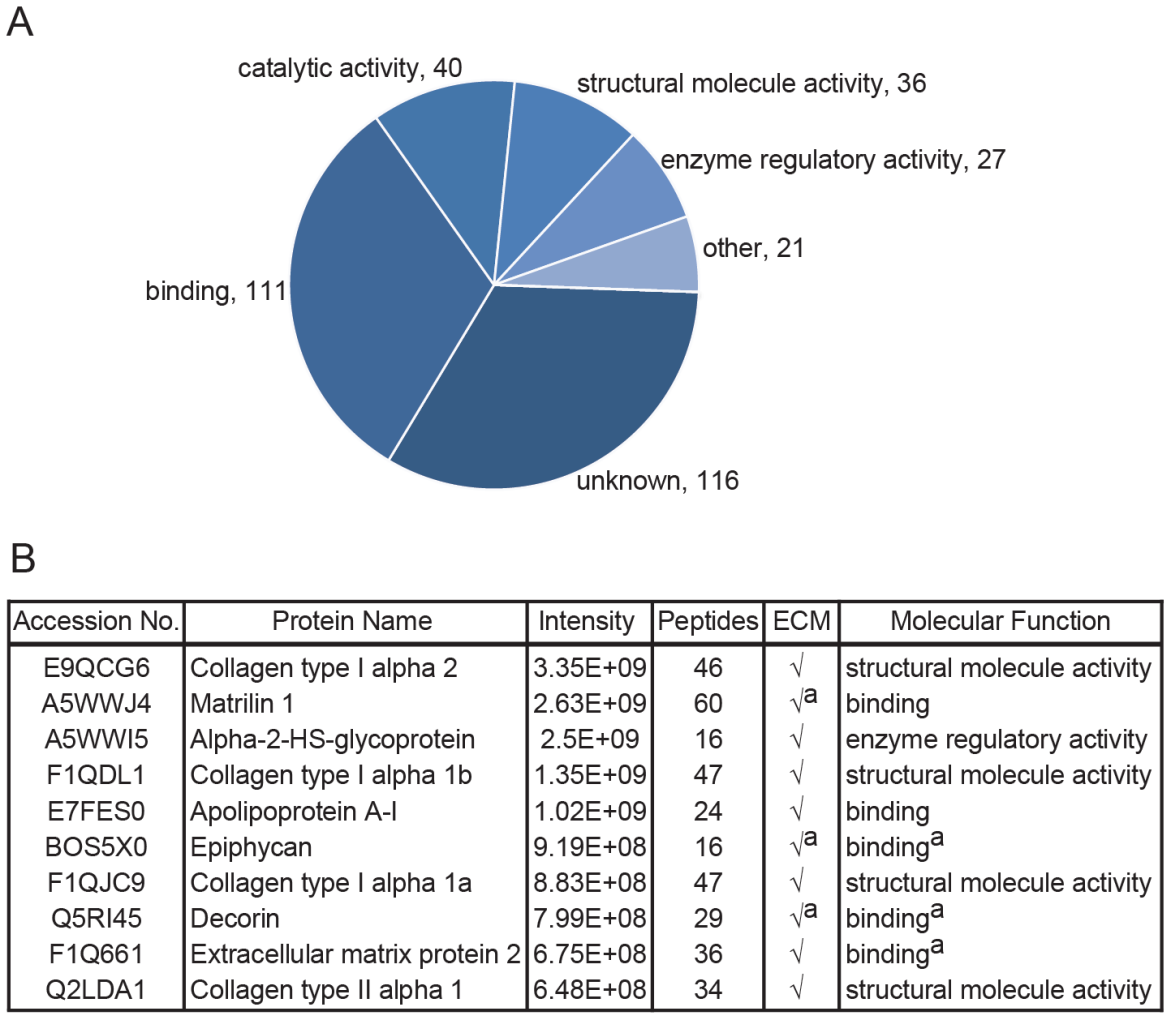
Composition of the zebrafish extracellular protein profile. (A) Distribution of molecular functions for the proteins identified as extracellular proteins. *^a^* Gene Ontology information based on the human ortholog of a protein in case this information was absent in the zebrafish database. The diagram contains 351 entries corresponding to 262 different proteins of which some fall into multiple categories (B) Ten most abundant extracellular proteins within the obtained protein profile based on their intensity.

**Figure 2:**
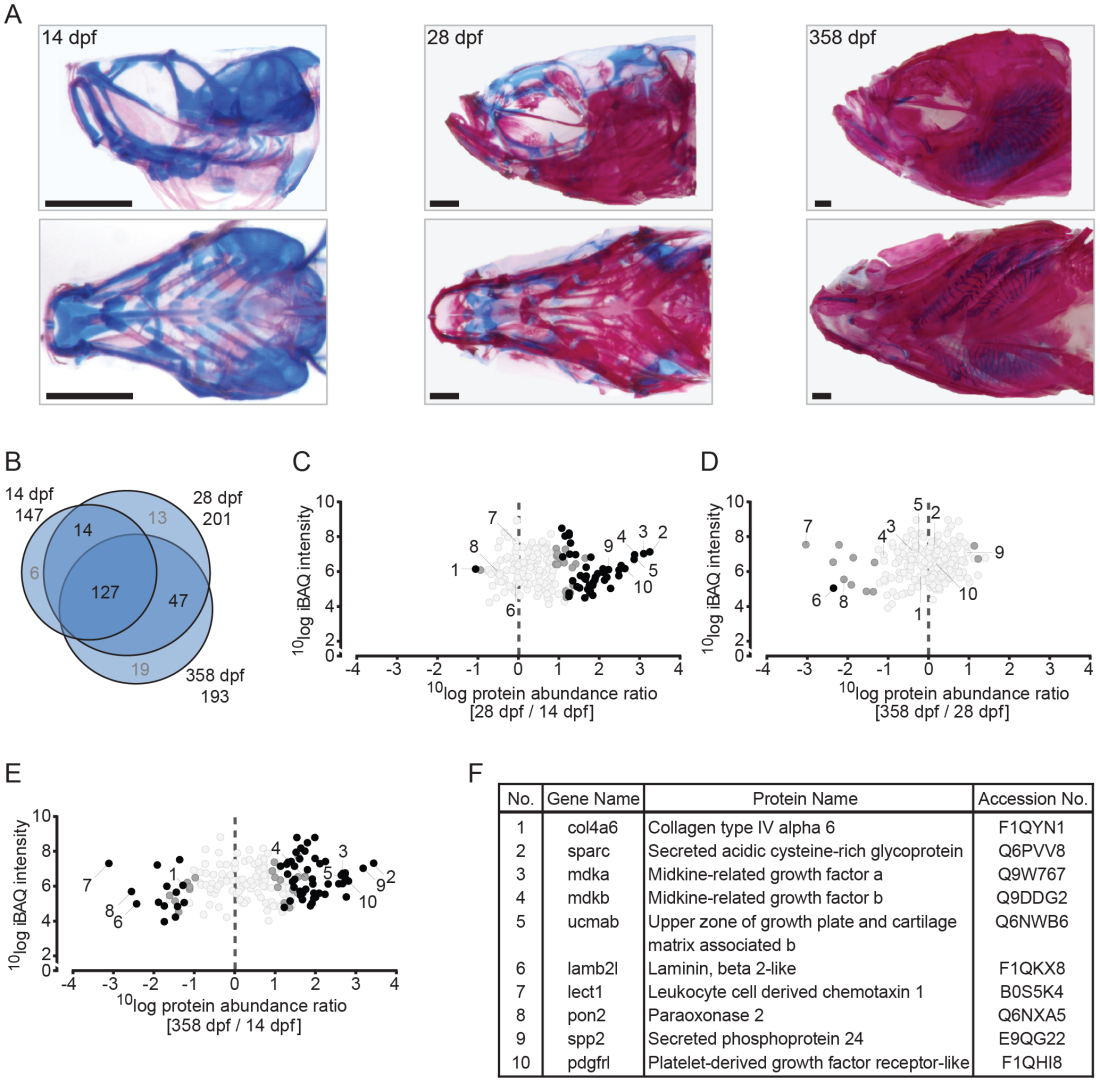
Quantitative analysis of the zebrafish craniofacial skeleton by MS-based proteomics. (A) Alcian blue/alizarin red stain of cartilage/bone structures in the craniofacial skeleton. Lateral (top) and ventral (bottom) images of all three time point used for protein extraction (left to right: 14 dpf, 28 dpf, 358 dpf). (B) VENN diagram of proteins selected for label-free quantification, with areas drawn to represent the number of proteins. Total number of proteins as well as distinct and common proteins are indicated for each time point. Proteins that did not qualify for label-free quantification are depicted in grey. (C-E) Ratio abundance plots showing log total iBAQ intensities versus log protein abundance ratio of the 28/14 dpf (C), the 358/28 dpf (D) and the 358/14 dpf (E) craniofacial skeleton ratios of all the proteins that met the strict criteria for label-free quantification (black circles, significantly differential abundant proteins at FDR =  0.01; dark grey circles, significantly differential abundant proteins at FDR =  0.05; light grey circles, no significant change in abundance). The numbers correspond to the proteins listed in 2F. (F) Table containing several of the significant differentially abundant proteins within the cranial skeleton. Specific proteins are discussed in the text.

**Figure 3:**
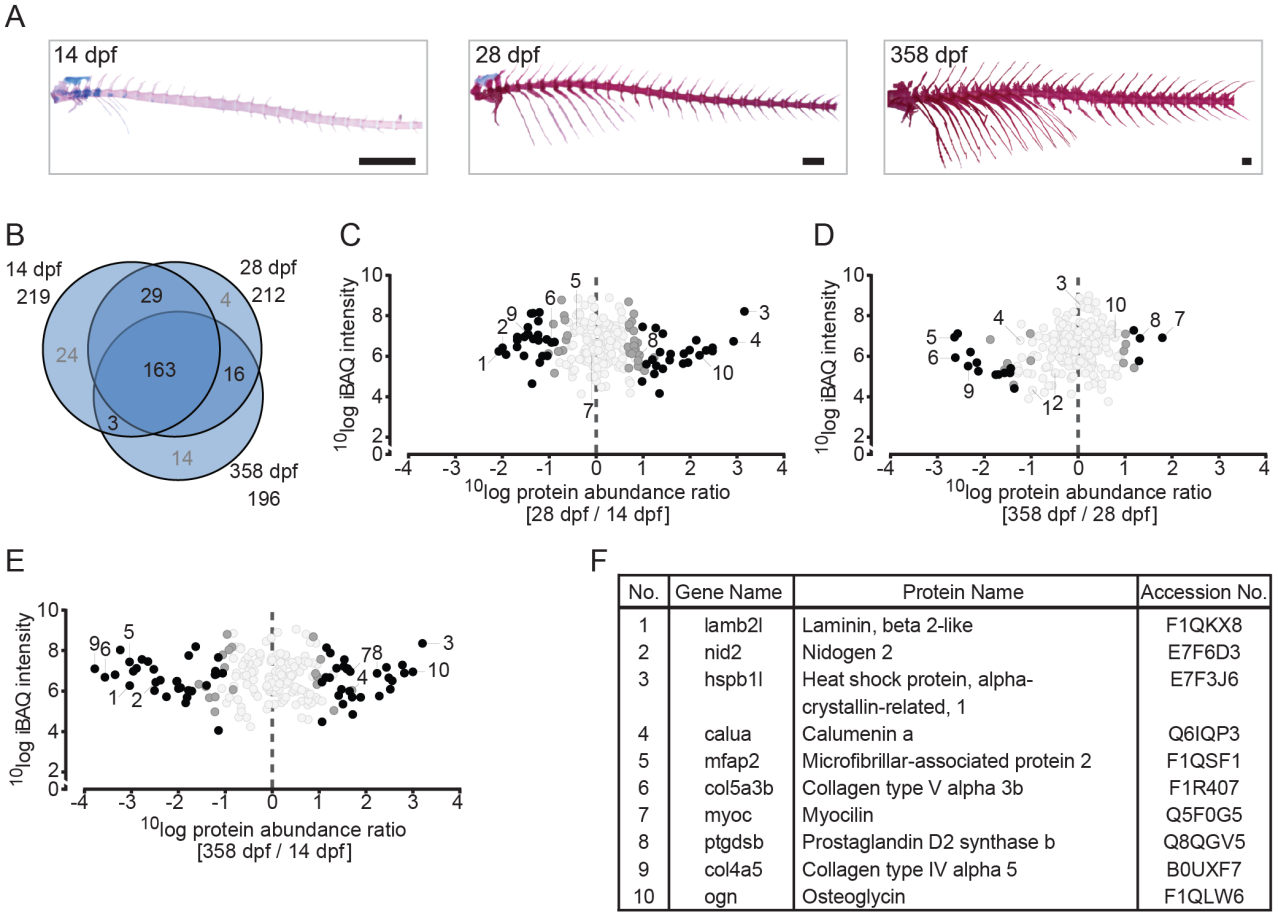
Quantitative analysis of the zebrafish axial skeleton by MS-based proteomics. (A) Alcian blue/alizarin red stain of cartilage/bone structures in the axial skeleton. Lateral images of all three time point used for protein extraction (left to right: 14 dpf, 28 dpf, 358 dpf). (B) VENN diagram of proteins selected for label-free quantification, with areas drawn to represent the number of proteins. Total number of proteins as well as distinct and common proteins are indicated for each time point. Proteins that did not qualify for label-free quantification are depicted in grey. (C-E) Ratio abundance plots showing log total iBAQ intensities versus log protein abundance ratio of the 28/14 dpf (C), the 358/28 dpf (D) and the 358/14 dpf (E) axial skeleton ratios of all the proteins that met the strict criteria for label-free quantification (black circles, significantly differential abundant proteins at FDR =  0.01; dark grey circles, significantly differential abundant proteins at FDR =  0.05; light grey circles, no significant change in abundance). The numbers correspond to the proteins listed in 3F. (F) Table containing several of the significant differentially abundant proteins within the axial skeleton. Specific proteins are discussed in the text.

**Figure 4:**
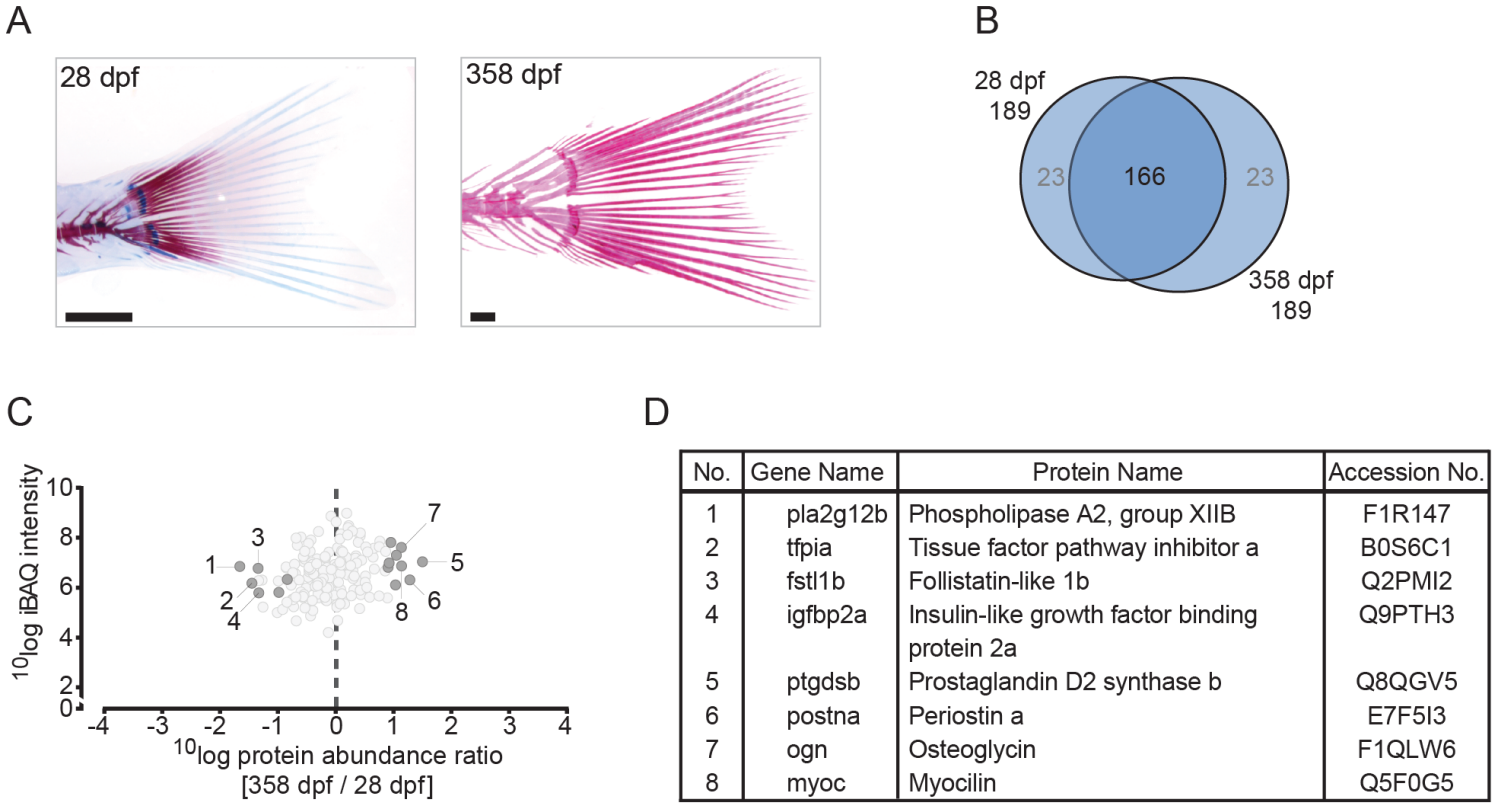
Quantitative analysis of the zebrafish caudal fin by MS-based proteomics. (A) Alcian blue/alizarin red stain of cartilage/bone structures in the caudal fin skeleton. Lateral images of two time points used for protein extraction (left to right: 28 dpf, 358 dpf). (B) VENN diagram of proteins selected for label-free quantification, with areas drawn to represent the number of proteins. Total number of proteins as well as distinct and common proteins are indicated for each time point. Proteins that did not qualify for label-free quantification are depicted in grey. (C) Ratio abundance plot showing log total iBAQ intensities versus log protein abundance ratio of the 358/28 dpf caudal fin region ratios of all the proteins that met the strict criteria for label-free quantification (dark grey circles, significantly differential abundant proteins at FDR =  0.05; light grey circles, no significant change in abundance). The numbers correspond to the proteins listed in 4D. (D) Table containing several of the significant differentially abundant proteins within the cranial skeleton. Specific proteins are discussed in the text.

A total of 262 different extracellular proteins (Fig. 1A) were identified and quantified in protein extracts of the zebrafish skeleton at three developmental stages of the craniofacial, axial and caudal fin regions (see Fig. 2, 3 and 4 respectively). Extracellular proteins were selected based on available GO information of the zebrafish (n =  126), and GO information based on human orthologs (n =  136) as described in the Materials and Methods section. Based on STRAP gene ontology analysis, the extracellular proteins were associated mainly with binding (n =  111), but also with processes like catalytic activity (n =  40), structural molecule activity (n =  36), and enzyme regulatory activity (n =  27) suggesting that next to major structural proteins, the selected protein set also included proteins with regulatory functions in skeletal development. A large number of proteins (n =  116) however lacked gene ontology information (Fig. 1A). The full list is shown in Table S1 in File S1 while an overview of all identified peptides is presented in Table S2 in File S1. One hundred and forty-eight cellular proteins were also identified but, together with proteins that could not be properly annotated, these were not further analyzed (Table S4 in File S1). As expected, the most abundant proteins within the list of extracellular proteins include major bone (e.g. collagen type I isoforms, extracellular matrix protein 2) and cartilage proteins (e.g. collagen type II, matrilin 1, apolipoprotein A-I, epyphican), previously described in MS based analyses of these tissues/structures in other vertebrate species, including humans [29], [30], [31], [32]. Also the serum-derived protein alpha-2-HS-glycoprotein (Ahsg), reported to bind to the mineral content of bone, was found among the most abundant proteins (Fig. 1B) [33], [34].

In addition to these highly abundant proteins, the total protein profile contained multiple other ECM structural constituents among which various types of collagens, as well as several proteins from the small leucine-rich proteoglycan (SLRP) family (e.g. biglycan, opticin, osteoglycin, asporin), growth factors (e.g. bone morphogenic proteins, transforming growth factors, fibroblast growth factors), growth factor regulator proteins (e.g., insulin-like growth factor binding proteins, transforming growth factor binding proteins), various peptidases (e.g. matrix metallopeptidases, serine peptidases, carboxypeptidases), peptidase inhibitors (e.g. serpin peptidase inhibitors, timp metallopeptidase inhibitors), and serum proteins (e.g., transferrin, thrombin, apolipoproteins, complement factors) (Table S3 in File S1).

### Analysis of extracellular matrix proteins during skeletal development

The relative contribution of cartilage and bone to the skeletal structures in the craniofacial sections changes rapidly (Fig. 2A). To identify extracellular matrix proteins associated with the different skeletal tissues during zebrafish development, relative quantification was performed separately for the craniofacial skeleton, the axial skeleton, and the caudal fin region of three developmental stages; larvae (14 dpf), juveniles (28 dpf) and adult fish (358 dpf). In total, 188 of the proteins identified in the craniofacial skeleton were selected for the relative quantification based analysis. Of these, 127 were found at all three time points (Fig. 2B) while limited numbers were found at two or only one time point. Normalized abundance ratios were compared between the three different stages at a threshold of FDR  =  0.01. Seventy-four proteins significantly differed in abundance between larval, juvenile and adult stages (Fig. 2C-E, Table S5 in File S1). Three different analyses are presented, providing a pair-wise comparison between the three developmental stages in terms of protein abundance compared with iBAQ intensity [35]. A selection of the proteins that differ most in abundance for the three different stages is given in Fig. 2F, and their positions indicated in the diagrams of Fig. 2C-E. Among the proteins strongly reduced in abundance upon development between two of the time points, collagen type IV alpha 6 (Col4a6), laminin beta 2-like (Lamb2l), leukocyte cell-derived chemotaxin 1 (Lect1) and serum paraoxonase/arylesterase 2 (Pon2) were found while a strong increase in abundance was noted for osteonectin (Sparc), midkine-related growth factor a (Mdka), midkine-related growth factor b (Mdkb), unique cartilage matrix-associated protein a (Ucmab), secreted phosphoprotein 24 (Spp2) and platelet-derived growth factor receptor-like (Pdgfr1). The entire list of craniofacial extracellular proteins, including the significantly differing proteins, is given in Table S5 in File S1.

A similar analysis is presented for the axial region (Fig. 3). There, 163 proteins were found to be present at all three time points and again a minority was found to be associated only with one or two time points. In comparing the most significant reduction or increase in protein abundance there are striking differences with the list shown for the head region. For example the proteins Lamb2l, Nidogen 2 (Nid2), microfibrillar-associated protein 2 (Mfap2), collagen type V alpha 3b (Col5a3b) and collagen type IV alpha 5 (Col4a5) show a reduced 358/14 dpf ratio. Although the analysis is limited to juvenile and adult stages of the caudal fin (Fig. 4A), due to the limited yield of skeletal material from the larvae, the list of proteins significantly changed in abundance is again different from the previous two. Of the 8 proteins differing in abundance between the juvenile and adult stages of caudal fin development (Fig. 4D), myocilin (Myoc) and prostaglandin D2 synthase b (Ptgdsb) were found to among the proteins that exhibited the highest difference in abundance in the axial protein abundance comparison at the same stages. None of the caudal fin proteins strongly differing in abundance are found in the list of protein abundance comparison of the head region.

Multiple cartilage matrix proteins, such as Lect1, hyaluronan and proteoglycan link protein 1b (Hapln1b) and opticin (Optc) were found to be reduced in abundance during development. This observation fits with the gradual replacement of cartilage by ossified structures during development. Other proteins including the collagen type IV isoform alpha 6 (Col4a6), Lamb2l, laminin beta 4 (Lamb4), and nidogen 1b (Nid1b) are conventional basement membrane proteins [36], [37]. The same proteins are also expressed by chondrocytes and in mice become less abundant during the aging process [38]. Other proteins were connected to either mineralization (ectonucleotide pyrophosphatase/ phosphodiesterase family member 1)[39], or remodeling of the bone matrix (annexin A2b) [40]. The extracellular protein slit homolog 3 (Slit3), and otospiralin (Otos) have not previously been associated with vertebrate skeletogenesis.

A number of extracellular proteins are highly abundant in the juvenile skeleton but not in the larval and adult stages, for example several Wnt signaling regulator proteins such as secreted frizzled-related protein 1b (Sfrp1b) and frizzled-related protein (Frzb). Both have a documented role in chondral ossification [41], [42] thereby corresponding with the process of chondral ossification which is most prominent at the juvenile stage, but no longer evident in the adult skeleton. Additional proteins most abundant at the juvenile stage in the head region include the cartilage protein ‘upper zone of growth plate and cartilage matrix associated b’ (Ucmab), as well as lysyl oxidase-like 2b (Loxl2b), and inter-alpha-trypsin inhibitor heavy chain family, member 6 (Itih6) previously identified in cartilage tissues during MS-based approaches [29], [30]. The protein tissue factor pathway inhibitor a (Tfpia) was the one extracellular protein considered as novel in vertebrate skeletogenesis that showed highest abundance at 28 dpf (Table S5 in File S1).

The remaining proteins were shown to increase during the complete developmental sequence analyzed (Table S5 in File S1). The list includes proteins with an implicated function in mineralization such as Ahsg and Spp2 [43], [44], in bone formation such as osteopontin (Spp1), and Sparc, but also in the process of cartilage maturation, including cartilage intermediate layer protein 2 (Cilp2), and tenascin Xb (Tnxb) [29]. Other proteins significantly increased in abundance included growth factor regulators such as fibroblast growth factor-binding protein 2 (Fgfbp2), and Insulin-like growth factor-binding protein 5b (Igfbp5b). This last protein has been implicated to be processed by the proteinase htra serine peptidase 1b (Htra1b) that is able to induce bone formation by regulating transforming growth factor beta (Tgfβ) signaling [45], but can also regulate IGF signaling by cleaving IGFBP5 [46]. From the list of 123 extracellular matrix proteins that were significantly differing in abundance (P =  0.01), 52 were novel in the context of zebrafish skeletal development or have only been studied based on their gene expression pattern by in situ hybridization at stages up to 5 dpf (Table 1).

**Table 1:** Extracellular matrix proteins novel in the zebrafish skeleton.

		Craniofacial skeleton			Axial skeleton			Caudal fin region	
Uniprot	Protein name	Ratio 28/14	Ratio 358/28	Ratio 358/14	Ratio 28/14	Ratio 358/28	Ratio 358/14	Ratio 358/28	References
**Bone-related proteins**									
F1QR30	Bgnb^a^	0.44	0.94	1.38**	0.02	0.51	0.54	0.73	[29], [30], [32]
Q803H7	Itm2ba^a^	1.48**	–0.27	1.21**	0.96*	0.02	0.98	–0.31	[48]
F1R0N0	Lrrc17^a^	1.72**	0.26	1.98**	0.14	–0.20	–0.06	–0.79	[49]
Q6NXA5	Pon2^a^	–0.47	–2.08*	–2.55**	–0.26	–1.69**	–1.96**	n.s.	[50]
B0S6K5	Tnw^a^	–0.49	0.03	–0.46	–1.23**	–0.55	–1.78**	–0.03	[51]
B0S525	Igfbp5b^a^	2.45**	0.28	2.73**	0.81*	0.15	0.95	–0.38	[31], [32], [52], [53]
F1RDU8	Fcgrt	0.73	–1.33*	–0.60	0.20	–1.35**	–1.15**	n.s.	[32]
E9QG22	Spp2	2.19**	0.96	3.15**	2.13**	0.30	2.43**	0.33	[44]
F1QXC6	Tgfb2l	1.14*	0.07	1.21*	1.42**	0.28	1.70**	0.01	[54], [55]
**Cartilage-related proteins**									
B7SDQ7	Ccdc80^a^	1.74**	0.15	1.89**	–0.06	0.28	0.21	0.00	[29]
F1Q775	Cilp^a^	0.48	–0.25	0.23	–1.24**	–1.29	–2.53**	n.s.	[29]
F1Q924	Col6a6^a^	–0.47	–0.51	–0.98*	–1.53**	0.32	–1.21**	0.06	[56]
F1QLW6	Ogn^a^	1.80**	0.86	2.66**	2.20**	0.79	3.00**	1.11*	[29], [30], [32]
F1QKL1	Thbs3a^a^	n.s.	n.s.	n.s.	0.72	0.34	1.06**	–0.93	[29]
F1R1P9	Thbs4b^a^	n.s.	n.s.	n.s.	0.56	0.91	1.47**	0.10	[29], [30]
E7F6W5	Cmn^a^	n.s.	n.s.	n.s.	–0.10	–2.29**	–2.39**	n.s.	[57]
F1RAC0	Aebp1 (2/2)	1.68**	0.53	2.21**	1.85**	0.66	2.51**	0.41	[29], [30]
F1Q5N7	Cilp2	1.16*	0.78	1.94**	0.54	0.60	1.14**	1.01*	[29], [58]
E7FB76	Col21a1	n.s.	n.s.	n.s.	–0.59	–1.26	–1.85**	n.s.	[30]
E7FF99	Fbn2	–0.10	–0.76	–0.86	–0.59	–0.56	–1.15**	–0.57	[29]
F1QLT3	Fgfbp2	1.50**	0.41	1.91**	0.26	–0.22	0.03	n.s.	[30]
F1R5Z2	Fndc1	1.80**	0.05	1.85**	1.24**	–0.48	0.76	–0.55	[59], [60]
F1Q5C3	Itih6	1.61**	–0.44	1.16	0.00	–0.55	–0.55	n.s.	[29]
E7F8X0	Srpx2	0.82	0.72	1.55*	0.83*	0.39	1.22**	0.88*	[29]
E7F1S4	Thbs2b	0.80	0.11	0.90	1.07**	–0.31	0.75	–0.75	[29]
E7EXE8	Tnxb	2.24**	0.50	2.74**	0.73	0.58	1.31*	0.56	[29]
**Bone- and cartilage-related proteins**									
Q804H0	Anxa1c^a^	–0.22	–1.52*	–1.74**	n.s.	n.s.	n.s.	n.s.	[29], [30], [32]
Q6IQP3	Calua^a^	n.s.	n.s.	n.s.	2.93**	–1.23	1.69*	–1.35	[29], [32]
Q5SPR2	Clu^a^	n.s.	n.s.	n.s.	1.58**	1.19**	2.77**	0.35	[29], [30], [32]
Q6AXL0	Cthrc1a^a^	1.92**	0.37	2.29**	n.s.	n.s.	n.s.	n.s.	[29], [32]
Q6NYE1	Fgb^a^	1.22**	–0.43	0.79	n.s.	n.s.	n.s.	n.s.	[29], [30], [32]
F1QG51	Fmoda^a^	–0.22	–0.32	–0.53	–1.21**	–0.42	–1.63**	–1.29	[29], [30], [32]
F1REI0	Htra3^a^	0.70	–1.09	–0.40	1.20**	–1.01*	0.19	–0.74	[61]
Q6GMI5	Bgna^a^	0.92*	0.67	1.59**	0.71*	0.52	1.23**	0.37	[30], [31], [32]
F1R6R2	Apcs	n.s.	n.s.	n.s.	–1.69**	–0.35	–2.04**	n.s.	[30], [32]
A9JRB3	Htra1b	1.64**	0.26	1.90**	0.68*	0.28	0.95*	–0.34	[62] [63]
F1QFZ8	Mmp13b	1.24**	0.07	1.31**	0.88*	0.72	1.61**	0.64	[64] [65]
F1QLH9	Nucb1	0.88	–0.13	0.75	1.36**	–0.93	0.43	–1.00*	[29]
Q29RB4	Olfml3a	0.69	0.60	1.29**	0.81*	0.55	1.37**	0.27	[29], [32]
B3DJG2	Pcolce (2/2)	1.62*	0.16	1.78**	2.48**	0.33	2.81**	0.32	[29], [30], [32]
**Novel proteins**									
F1QW52	Habp2	1.62**	0.04	1.67**	–0.22	–0.32	–0.54	–0.31	–
E7F8L7	Otos	0.28	–2.20*	–1.91**	–0.68*	–2.56**	–3.24**	n.s.	–
F1Q9N5	Metrnl	n.s.	n.s.	n.s.	1.28**	–0.34	0.94	–0.62	–
B0UXN0	Qsox1	n.s.	n.s.	n.s.	0.98**	1.30**	2.28**	n.s.	–
F1QK57	Tecta	1.10	0.96	2.07**	n.s.	n.s.	n.s.	n.s.	–
B0S6C1	Tfpia	1.85**	–0.67	1.18	0.92*	–0.86	0.06	–1.46*	–

Of the 123 extracellular proteins that were differentially abundant during zebrafish skeletal development, a subset of 40 proteins was identified as novel proteins in zebrafish skeletal development based on whether they were previously characterized or identified in skeletal tissues of other vertebrate species (mouse, rat, human). In addittion, 6 were considered as novel in vertebrate skeletogenesis in general (bottom). ^a^ Indicates proteins previously studied based on their gene expression pattern by in situ hybridization in stages up to 5 dpf. Proteins that were not selected for relative quantification based on described criteria (Materials and Methods section) are marked (N.S.). ^*^ Significantly differential abundant proteins based on their significance at a threshold of FDR =  0.05. ^**^ Significantly differential abundant proteins based on their significance at a threshold of FDR =  0.01.

Finally, a small number of extracellular proteins were found that can be regarded as novel in the context of vertebrate skeletogenesis in general. This list includes tissue factor pathway inhibitor a (Tfpia), hyaluronan binding protein 2 (Habp2), otospiralin (Otos), tectorin alpha (Tecta), meteorin, and glial cell differentiation regulator-like (Metrnl). While there is no clear picture emerging from this small list of proteins in relation to the signaling processes or the structural proteins involved in bone formation, there seem to be a number of proteins acting as growth inhibitors or representing elements of other tissues such as blood vessels or neurons. For instance, the protein Metrnl is known to act on glial cell differentiation of mice [47].

**Figure 5:**
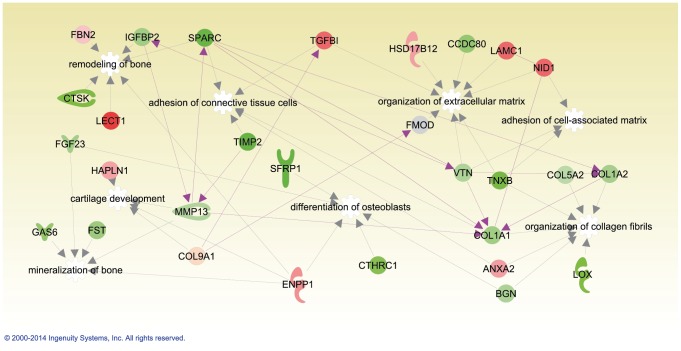
Ingenuity Pathway Analysis (IPA). Proteins that differ in abundance are involved in connective tissue, skeletal and muscular development. Green shading indicates an increase in abundance during development whereas red shading indicates a decrease. Increased intensity in colors indicates a higher differential abundance.

The obtained protein abundance ratios were used for analysis using the Ingenuity Pathway Analysis (IPA) tool. As shown in Fig. 5, the network consisting of 29 proteins is enriched in proteins linked to processes that contribute to formation of the extracellular matrix, including cell-matrix interactions, organization of collagen type I fibrils, mineralization of bone, and bone remodeling. During development, proteins contributing to the cartilage matrix (e.g. Hapln1) decreased in abundance whereas most proteins involved in the formation and remodeling of bone increased.

**Figure 6:**
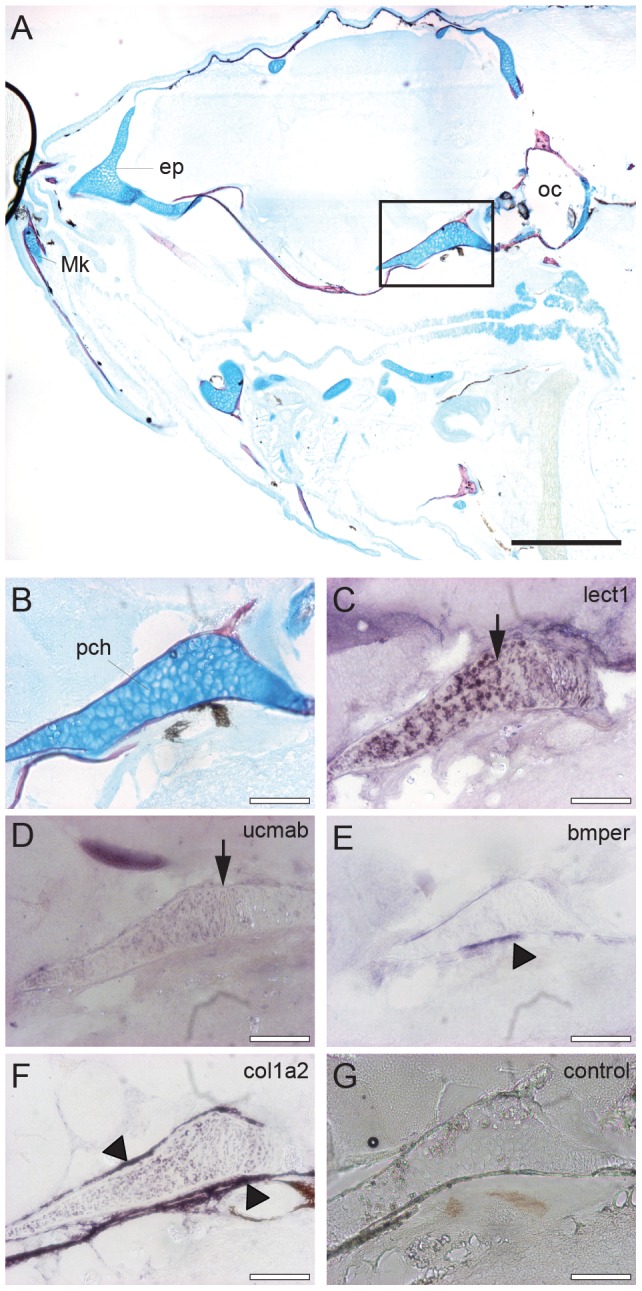
In situ mRNA hybridization of genes encoding extracellular proteins implicated in cartilage and bone formation in the head region. Sagittal sections of the head region of 28 dpf zebrafish juveniles stained by acid-free bone (red) and cartilage (blue) staining (A-B), or used for in situ hybridization with an antisense RNA probe corresponding to lect1 (C), ucmab (D), bmper (E), col1a2 (F). As a control, hybridization with the lect1 sense RNA probe is shown (G). (B-G) Magnification of boxed area in A focusing on the parachordal (pch) cartilage. Images are from consecutive sections at the same position. (C-D) Expression of lect1 and ucmab is located in chondrocytes of the parachordal (pch) cartilage (arrows). (E-F) Transcripts of bmper and col1a2 were detected in ossification sites surrounding the parachondral chondrocytes (arrow heads). Scale bars indicate 0.5 mm (black), or 100 µm (white). Abbreviations: ep, ethmoid plate; Mk, Meckel’s cartilage; oc, otic capsule; pch, parachordal.

To determine whether the extracellular proteins as identified here are secreted by either chondrocytes or osteoblasts, in situ mRNA hybridization was employed for the cartilage-related genes lect1 and ucmab and the bone-related genes bmper and col1a2 (Fig. 6). Sagittal sections of the head region of 28 dpf zebrafish juveniles were used, as both cartilage and bone formation are actively taking place at this stage (cf. Fig. 2A). The results show that lect1 and ucmab expression is found in chondrocytes of the parachordal (pch) cartilage (arrows) whereas bmper is expressed in ossification sites as confirmed by col1a2 expression. This confirms that these extracellular proteins are deposited close to their respective sites of secretion.

## Discussion

In order to obtain a more comprehensive understanding of the factors that are pivotal during osteogenesis and in bone function, it is important to determine the protein composition of the osteoid matrix during earlier and more mature stages of bone formation. This is technically challenging, and a systematic analysis of proteins at different stages of osteogenesis has for this reason has not yet been carried out previously. In this study, we present an overview of the extracellular proteins present in zebrafish bone and cartilage elements at three different stages, as determined by LC-MS/MS.

As expected, major bone and cartilage constituents such as collagen type I and collagen type II were among the most abundant proteins. Less abundant proteins, including most of the described non-collagenous proteins in bone (e.g. Spp1, Sparc), multiple proteoglycans including almost the complete small leucine-rich protein family (e.g. decorin, biglycan), and even serum proteins described to bind to the bone mineral content (e.g. Fetub, Ahsg) were detected [33], [34], [43]. Our results indicate that most of the intracellular proteins were significantly reduced and this supports the validity of the procedures that have been employed for tissue purification and protein extraction. In comparison to the previous study describing 417 proteins in zebrafish caudal fins [66], few correspondences were found. Out of the 19 proteins found corresponding with the previous study, only 3 (Anxa1b, Col12a1 and Col6a1) were isoforms of the ones in our list of proteins novel to zebrafish bone and cartilage formation (Table 1). This apparent lack of correspondence is most likely due to the inclusion of the full set of intracellular proteins in the previous study [66].

To obtain an overview of major differences in skeletal composition during development, 262 extracellular proteins were detected and compared at three different stages of development; larval, juvenile, and adult. 123 differentially abundant proteins were identified, among which were well-established components of the vertebrate skeleton. Among these, 40 extracellular proteins were considered as not previously connected to zebrafish skeletogenesis. Based on functional analysis in other vertebrate species, most of the new entries corresponded with major stage-specific differences such as the initial growth of cartilage elements followed by the ossification of most of these elements, and the increase of bone matrix by a growing number of bone structures together with an overall increase in the degree of mineralization. An unexpected finding was that the largest changes in protein abundance were highly dissimilar in the head, axis and caudal fin regions. This may be the result of underlying temporal or spatial differences in skeletal organization between the three regions and underscores the necessity to incorporate detailed localization studies in a proteomics approach such as performed here.

One of the here identified novel proteins in zebrafish skeletogenesis was the protein Cilp2. This protein is believed to be a component of permanent cartilages in mice, since it is not observed in replacement cartilage [58]. In the zebrafish, the ossification of elements in the skull is completed around 70 dpf. In this study, the protein Cilp2 was significantly increased in the adult skeleton, at which point replacement cartilages are no longer present in the zebrafish skeleton. Permanent cartilages in the zebrafish are mainly observed in later stages of development in the skull in which cartilage bands connect several bones, and between the individual vertebrae [67]. The absence of replacement cartilage in the adult zebrafish, and the here observed up-regulation of Cilp2 in the adult skeleton therefore suggests that in zebrafish, Cilp2 also contributes to permanent cartilages.

Another interesting finding was the significant up-regulation of the Wnt signaling pathway antagonists, Frzb and Sfrp1b at the juvenile stage during zebrafish skeletal development. In mice, FRZB is implicated in skeletal development by modulating chondrocyte maturation and preventing chondrocytes from entering the hypertrophic phase during perichondral ossification [42]. Since perichondral ossification in the zebrafish is observed in the larval and juvenile craniofacial skeleton, but is no longer evident in the adult, the up-regulation of Frzb at the juvenile stage is correlating with this process. The endogenous Wnt signaling antagonist SFRP1 is also implicated in this process by regulating the Wnt/β-catenin signaling pathway, affecting chondrocyte maturation and ossification events in the cranial base of mice [68]. The involvement of the Wnt signaling pathway in zebrafish cartilage morphogenesis has already been shown [69]. Together with the fact that Wnt proteins are often highly conserved across species [70], the zebrafish could well be used as a model to elucidate the signaling pathways contributing to the formation of skeletal elements.

The effects of the insulin-like growth factor system on skeletal development and maintenance has already been subjected to intensive studies [71], [72]. Insulin-like growth factor inhibitors, like the here identified Igfbp5b have been implicated in either maintaining osteoblasts in an immature status, or promoting osteoclastogenesis and subsequent bone resorption [73]. In line with this, increase in human IGFBP5 has been linked to age-related bone loss via an increased rate of bone-resorption [52]. Here we identified a significant increase in Igfbp5b abundance in the adult zebrafish skeleton, when compared to younger stages. The combined significant increase in Igfbp5b and collagenase 3 (Mmp13b) emphasizes the possible increase in osteoclastogenesis. Mmp13b is involved in bone resorption but also in the differentiation of osteoclasts [65], [74]. The increase of both Igfbp5b, and Mmp13b implicates an increase in osteoclast differentiation and/or activity in adult zebrafish compared to younger developmental stages. The use of transgenic lines that allow visualization of osteoclasts *in vivo* will be a tremendous tool to correlate protein distribution with osteoclast activity in the future [75].

The formation of bone is a gradual process during which proteins such as Sparc are embedded during development [76]. In this study, most bone related proteins were found to increase during the developmental analysis of the ECM proteins. Only one extracellular protein with a function in mineralization was significant decreased in abundance during development. This protein, ectonucleotide pyrophosphatase/ phosphodiesterase family member 1 (Enpp1) is a known regulator of phosphate/pyrophosphate homeostasis *in vivo* [39]. Since pyrophosphate is a strong chemical inhibitor of bone mineral formation, the decrease of Enpp1 during development correlates with the progression of ossification observed during the formation of the zebrafish skeleton.

In summary, this study demonstrates that the secreted proteome reflects all major processes taking place during skeletal development of the zebrafish. Processes including cartilage proliferation and morphogenesis, gradual ossification, and even several signaling pathways based on secreted growth factors and growth factor regulators were identified. The substantial number of extracellular zebrafish proteins identified here, which have a mammalian ortholog previously associated with osteogenesis, underscores the validity of our MS-based approach, and we demonstrate that the composition of the zebrafish extracellular matrix has striking similarities to that of other vertebrate species, including mammals. Significantly, we furthermore also identify a number of proteins that have not been connected to osteogenesis previously. These proteins warrant further analysis, and will serve as a very useful reference for future studies not only in zebrafish, but also in other vertebrate species.

## Supporting Information

Figure S1 **Histological analysis of the zebrafish larval, juvenile and adult skeleton.** Lateral view of skeltal elements of (A-D) zebrafish larve, (E-H) juvenile, and (I-L) adult stage as revealed by acid-free bone and cartilage double staining. (B, F, J) Magnification of the zebrafish skull region. (C,G,K) Magnification of the first three caudal vertebrae. (D, H, L) Magnification of the caudal fin region. Scale bars indicate 1 mm (black), or 0.25 mm (white). Abbreviations: bsr, branchiostagel rays; cb, ceratobranchial; cl, cleithrum; ep, epural; f, frontal; fr, fin rays; ha, haemal arch; hm, hyomandibula; hprez, haemal prezygapophyses; hpstz, haemal postzygapophyses; hs, haemal spine; hspu, haemal spine of preural; hy, hypural; Mk, Merckel’s cartilage; na, neural arch; nprez, neural prezygapophyses; npstz, neural postzygapophyses; ns, neural spine; nspu, neural spine of preural; phy, parhypurals; ts, tectum synoticum.
(TIF)

File S1 **File S1 includes the following: Table S1.** Original protein groups table as obtained from the MaxQuant software. **Table S2.** Original peptides table as obtained from the MaxQuant software. **Table S3.** Identified Extracellular proteins. GO annotations obtained by STRAP analysis on zebrafish proteins and human orthologue proteins. **Table S4.** Cellular proteins and proteins without GO annotations. **Table S5.** Differential protein abundance. **Table S6.** In situ hybridization primer sequences of genes from the selected proteins.
(XLSX)
