# Supplementary figures and images for: Proteomics Analysis of the Zebrafish Skeletal Extracellular Matrix
Source: PLoS One. 2014 Mar 7;9(3):e90568. doi: 10.1371/journal.pone.0090568 (PMC3946537; doi:10.1371/journal.pone.0090568)

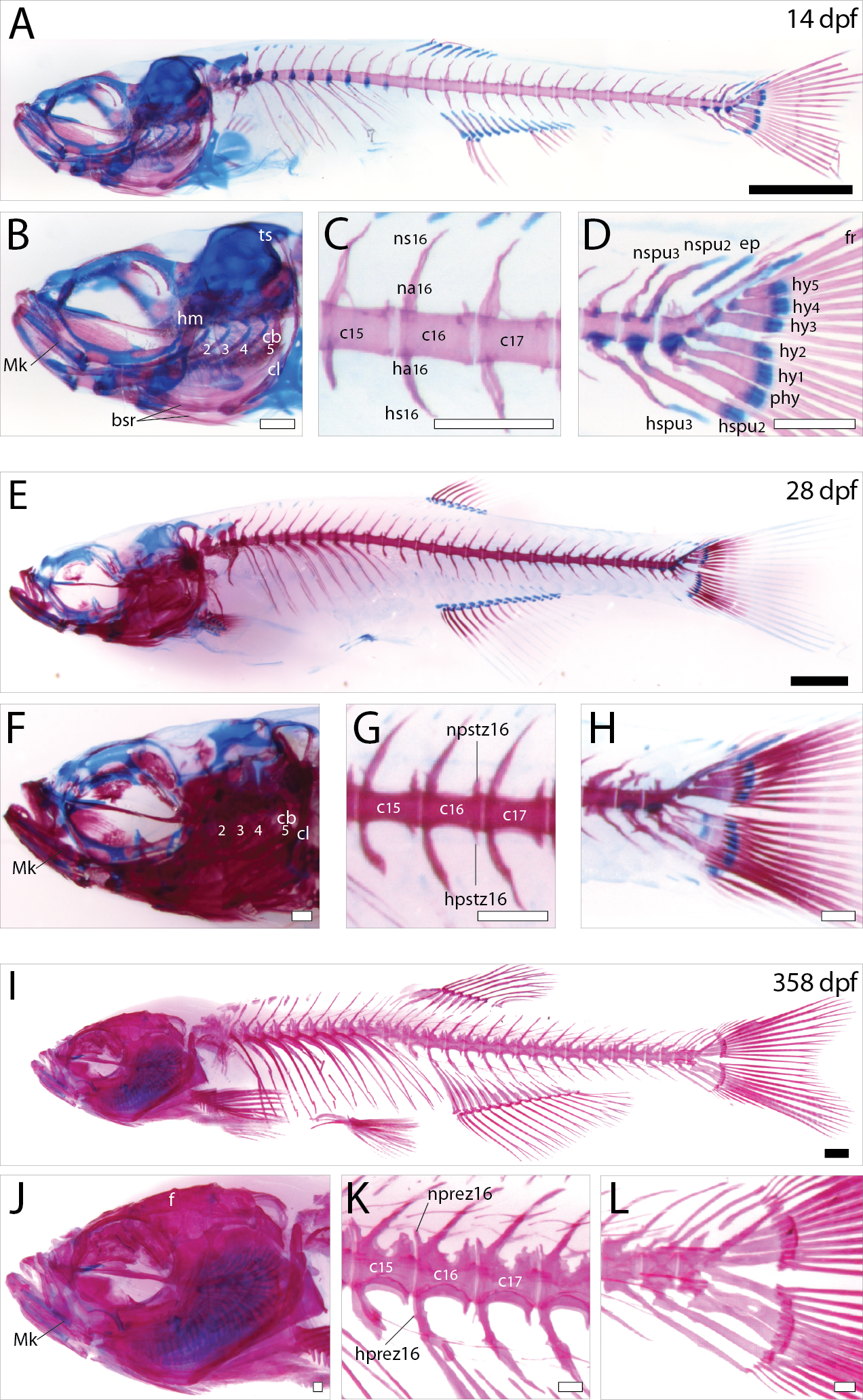

Supplement: Figure S1 — Histological analysis of the zebrafish larval, juvenile and adult skeleton. Lateral view of skeltal elements of (A-D) zebrafish larve, (E-H) juvenile, and (I-L) adult stage as revealed by acid-free bone and cartilage double staining. (B, F, J) Magnification of the zebrafish skull region. (C,G,K) Magnification of the first three caudal vertebrae. (D, H, L) Magnification of the caudal fin region. Scale bars indicate 1 mm (black), or 0.25 mm (white). Abbreviations: bsr, branchiostagel rays; cb, ceratobranchial; cl, cleithrum; ep, epural; f, frontal; fr, fin rays; ha, haemal arch; hm, hyomandibula; hprez, haemal prezygapophyses; hpstz, haemal postzygapophyses; hs, haemal spine; hspu, haemal spine of preural; hy, hypural; Mk, Merckel’s cartilage; na, neural arch; nprez, neural prezygapophyses; npstz, neural postzygapophyses; ns, neural spine; nspu, neural spine of preural; phy, parhypurals; ts, tectum synoticum. (TIF) [file pone.0090568.s001.tif]
